# Effect of Third-Stage Heat Treatments on Microstructure and Properties of Dual-Phase Titanium Alloy

**DOI:** 10.3390/ma14112776

**Published:** 2021-05-24

**Authors:** Xiqin Mao, Meigui Ou, Desong Chen, Ming Yang, Wei Long

**Affiliations:** 1 College of Materials and Metallurgy, Guizhou University, Guiyang 550025, China; Xiqin_Mao@163.com (X.M.); cds271828@163.com (D.C.); longwei3810@163.com (W.L.); 2 Key Laboratory for Materials Structure and Strength of Guizhou Province, Guizhou University, Guiyang 550025, China

**Keywords:** β phase zone, third-stage heat treatments, TC21 titanium alloy, microstructure, properties

## Abstract

Two-phase TC21 titanium alloy samples were solution-treated at 990 °C (β phase zone) and cooled by furnace cooling (FC), air cooling (AC), and water quenching (WQ), respectively. The second solution stage treatment was carried out at 900 °C (α + β phase zone), then aging treatment was performed at 590 °C. The influence of the size and quantity of the α phase on the properties of the sample were studied. The experimental results showed as the cooling rate increased after the first solution stage treatment, wherein the thickness of primary layer α gradually decreased, and the tensile strength and yield strength gradually increased. After the second solution stage treatment, the tensile properties of samples increased due to the quantity of layers α increased. The aging treatment promoted the precipitation of the dispersed α phase and further improved the tensile strength. After the third solution stage treatments, the FC samples with more β-phase had the best comprehensive mechanical properties.

## 1. Introduction

TC21 titanium alloy is widely used in aerospace and automotive fields due to its excellent mechanical properties and corrosion resistance [1,2,3]. An Al element in TC21 alloys can enhance the strength at room and high temperature by forming a replacement solid solution. Mo, Nb, and Cr elements can increase the hardenability so as to improve the strength. Sn and Si elements make the alloy have high strength and heat resistance [4,5]. Heat treatment has an important influence on the mechanical properties of titanium alloys. At present, the commonly used heat treatment process to improve the comprehensive mechanical properties of titanium alloys is the second-stage heat treatment process (solid solution + aging) [6].

Wang [7] studied the effects of solid solution temperature on the phase transformation of TC21 titanium alloy and found that martensite appeared when the solid solution temperature was higher than 840 °C and then WQ. R. Filip [8] research results showed that as the cooling rate and β content increased, the thickness and length of the α phase decreased. Liu [9] studied the effects of different heat treatments on the microstructure and mechanical properties of TC4 alloy. After 960 °C/1 h with WQ and 500 °C/4 h with AC, a basket structure with finer recrystallized grains could be obtained, which improved the comprehensive mechanical properties. Wang [10] studied that TC21 titanium alloy is mainly composed of equiaxed grains and residual phases, and 880 °C is the appropriate annealing temperature for uniform microstructure refinement of hot-rolled TC21 titanium alloy. Hou [11] studied the effects of solution cooling rate and aging temperature on the structure of TC21 alloy. The results showed that the morphology of the primary α phase was mainly affected by the solid solution, and different cooling rates significantly affected the volume ratio of β phases and secondary α phases.

The ratio of equiaxed, lamellar, and dispersed-distributed α phases can be effectively controlled by three-stage heat treatments, compared with the single structure after double treatments. Li [12] studied the equiaxed, lamellar, and needle-like α ternary structure of TA19 titanium alloy after triple heat treatment. Chi [13] adopted three-stage heat treatment. The microstructure and the corresponding mechanical properties of Ti-Al-V-Mo-Zr alloy tubes were studied. The results show that the impact toughness is significantly improved, and the coarse equiaxial and lamellar structures are formed. Shi [14] studied the microstructure and mechanical properties of TC21 alloy after near-isothermal forging with different parameters plus solution treatment and aging. After aging at 590 °C for 4 h (third-stage), a fine α phase was obtained on the remained β phase. Both the tensile strength and the yield strength decreased with the increase of the coarse α-phase quantities. The decrease of the effective slip length and the improvement of the crack growth resistance improved the plasticity.

In this work, third-stage heat treatments were performed on the TC21 alloy and studied the effects of different cooling methods after the first solution stage treatment on the morphology and mechanical properties of samples. The results provide an idea for the improvement of the mechanical properties of the two-phase titanium alloy.

## 2. Materials and Methods

**Figure 1:**
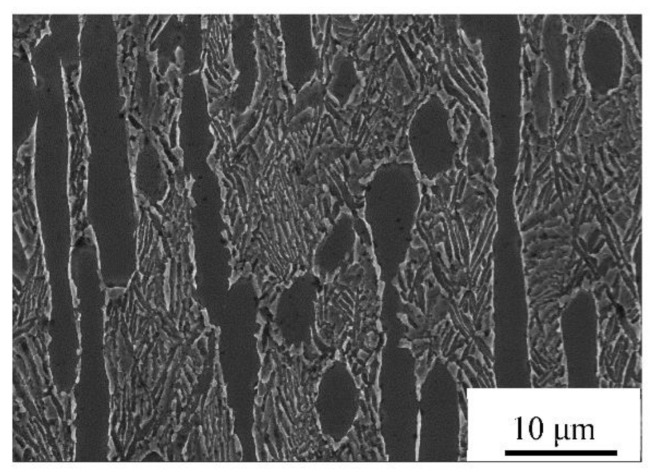
Original microstructure of TC21 samples.

The original material of TC21 alloy was provided by Northwest Institute of Nonferrous Metals. The phase transition point of TC21 alloy is 975 ± 5 °C. The chemical composition (mass fraction, %) is as follows: Al: 6.47, Zr: 2.28, Sn: 2.18, Mo: 3.23, Nb: 2.11, Cr: 1.51, Si: 0.11, Ti: Bal. The original microstructure of the forged and annealed material is presented in Figure 1.

**Figure 2:**
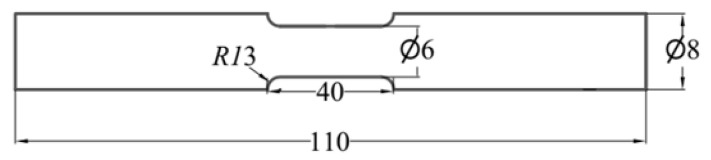
Schematic diagram of static tensile sample (mm).

**Figure 3:**
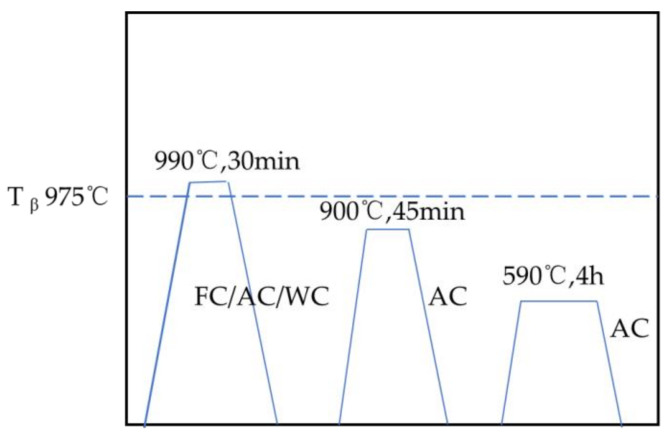
Heat treatment process.

According to the national standard (GB/T228-2002), the tensile samples were shaped to ∅6 mm × 110 mm with the gauge length of 30 mm in Figure 2; the impact samples was designed as the standard Charpy V-shaped notch (10 × 10 × 55 mm^3^), with a notch depth of 2 mm and a notch radius of 0.25 mm. The samples were treated by a first solution stage (990 °C, 30 min, FC/AC/WQ) in a β-phase zone. Based on previous work [15], the samples obtained at different cooling rates were subjected to a second solution stage (900 °C, 45 min, AC) and finally to aging treatment (590 °C, 4 h, AC). The heat treatment process is shown in Figure 3.

The samples were grinded with 80-7000# sandpaper and then polished to the mirror surface. A mixture of HF:HNO_3_:H_2_O (1:2:5 in volume ratio) was used to corrode the metallographic samples for 3–4 s. The microstructure and fracture morphology were observed via field-emission scanning electron microscopy (FESEM, Supra 40, Carl Zeiss AG, Oberkochen, Germany) with an acceleration voltage of 15 kV. The volume fraction of α after heat treatment at different temperatures was measured by software Image-Pro Plus, and the data were the average result of 20 images. Tensile properties were carried out by a universal testing machine (MTS Landmark, MTS System Co., Ltd., Eden Prairie, MN, United States) at a 1 mm/min rate. Impact experiments were tested on a pendulum instrumented impact testing machine (NI300C, NCS Testing technology Co., Ltd., Beijing, China). Three samples of each condition were investigated and tested.

## 3. Results and Discussion

**Figure 4:**
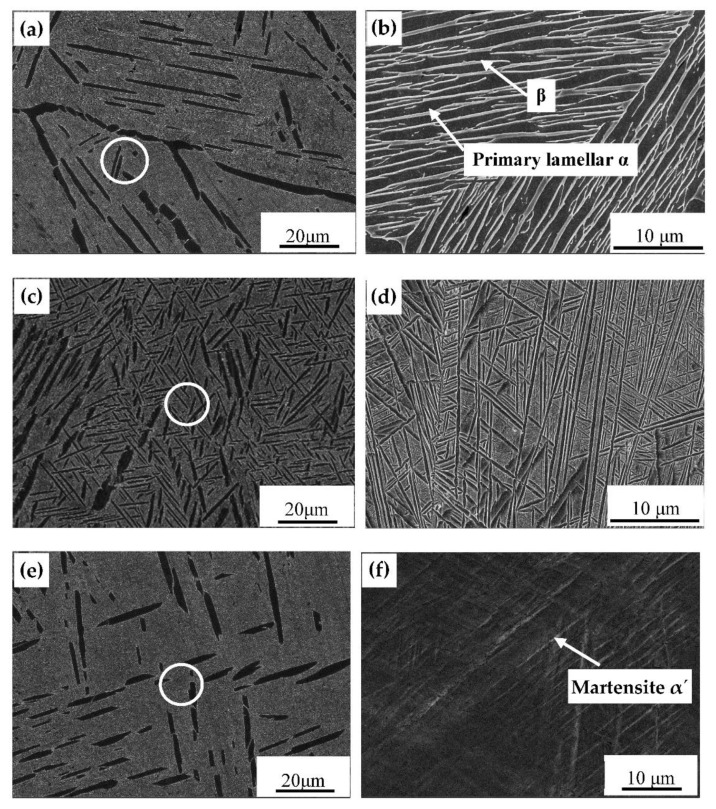
SEM images of microstructure of samples after the first solution stage treatment (**a**,**b**) FC; (**c**,**d**) AC; (**e**,**f**) WC.

The microstructure of the samples after the first solution stage processes is shown in Figure 4, and the thickness and volume percentage of primary lamellar α phases after heat processes are shown in Table 1. With the increase of cooling rate (FC< AC < WQ), the thickness of lamellar α phase decreased gradually [16]. Compared to the WQ, the cooling rate of FC (Figure 4a,b) and AC (Figure 4c,d) were relatively slower, and the degrees of supercooling were smaller. Therefore, the elements in the sample were adequately diffused, which benefited the growth of the lamellar α phase. As a result, obvious α colonies were observed in the furnace-cooled sample (Figure 4a,b), rather than the water-quenched sample (Figure 4e,f), which was consistent with the study results of Lutjering [17].

Moreover, a small amount of transformed β phase can be seen in Figure 4d due to the low cooling rate of AC. In the case of WQ, the fast-cooling rate and the large degree of supercooling led to the inhibition of diffusion of alloy elements and the occurrence of martensitic transformation [18,19]. The α phase was distributed in the β matrix in a fine needle shape, forming a supersaturated solid solution. This indicates that the cooling rate after the first solution stage treatment controlled the morphology and size of the primary lamellar α.

**Figure 5:**
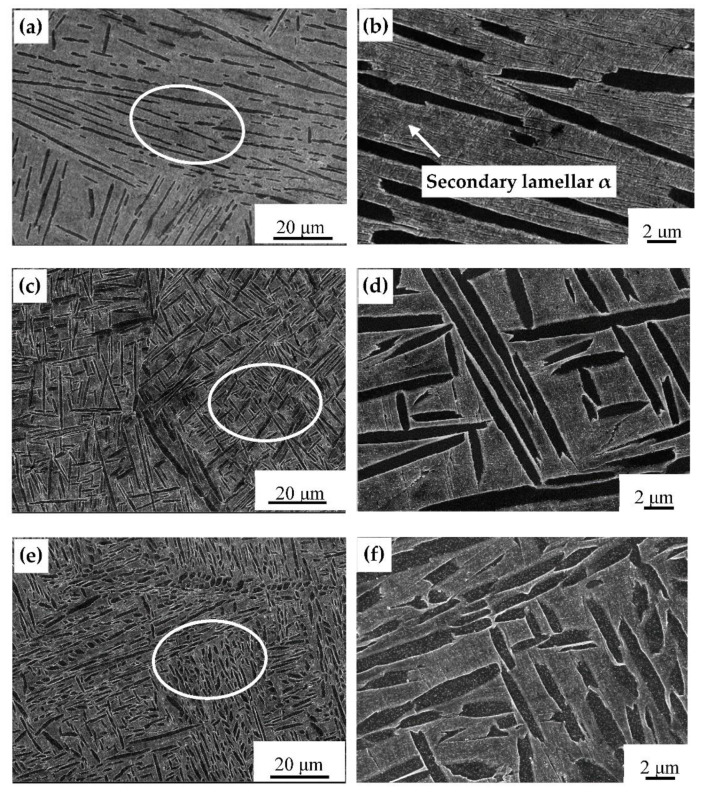
SEM images of microstructures of samples after the second-stage solution treatment (**a**,**b**) FC; (**c**,**d**) AC; (**e**,**f**) WQ.

Figure 5 shows the microstructure of the samples after the second solution stage treatment. It can be seen that the thickness of the primary layer α increased compared to the samples after the first solution stage treatment. After FC (Figure 5a,b) and AC (Figure 5c,d), the lamellar α phases remained in the samples, but no longer formed a cluster shape, and more lamella secondary α phases were precipitated from the β phases and staggered on the β matrix [20]. A small number of secondary α phases were also precipitated in the β phases after WQ (Figure 5e,f), and their interlacing degree increased significantly. The final microstructure consisted of a primary lamellar α and β phase. As the cooling rate increased (FC < AC < WQ), the quantity of secondary α phases decreased. Due to the fast-cooling rate of WQ in the first solution stage treatment, more metastable β phases were retained and provided favorable conditions for the transformation of β phase into primary α phases. This result indicates that the second solution stage treatment mainly controlled the morphology and size of the secondary lamellar α.

**Figure 6:**
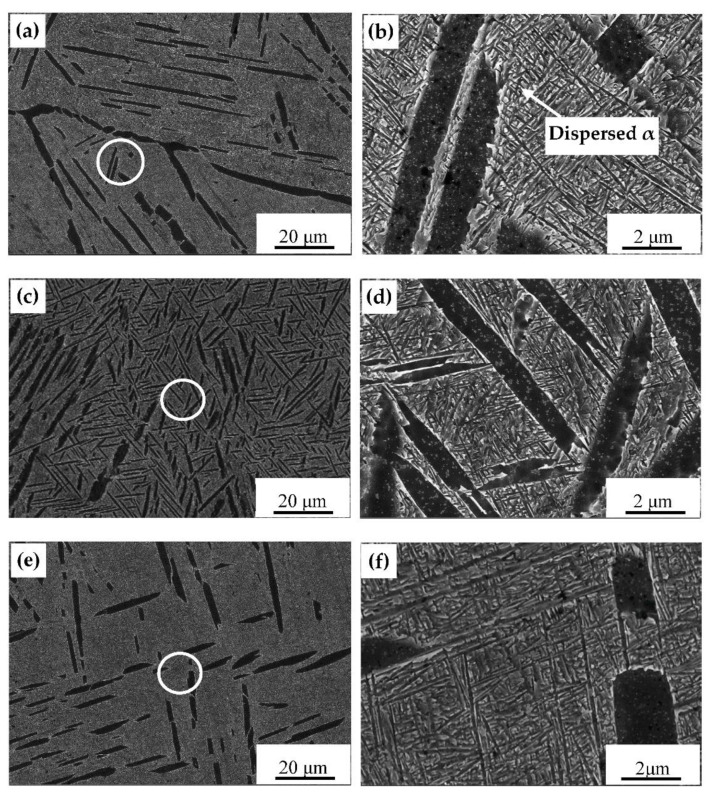
SEM images of microstructures of samples after aging treatment (**a**,**b**) FC; (**c**,**d**) AC; (**e**,**f**) WQ.

The microstructure of the samples after the third solution treatment are shown in Figure 6. It can be observed that the quantity of the primary lamellar α phase of the samples decreased, and a large number of dispersed α phases formed in the β matrix because the α phase had sufficient energy for nucleation and precipitation during the aging process. Moreover, the quantity of primary lamellar α decreased, whereas the amount of secondary lamellar α increased, which was consistent with the study results of Yu et al. [21].

**Figure 7:**
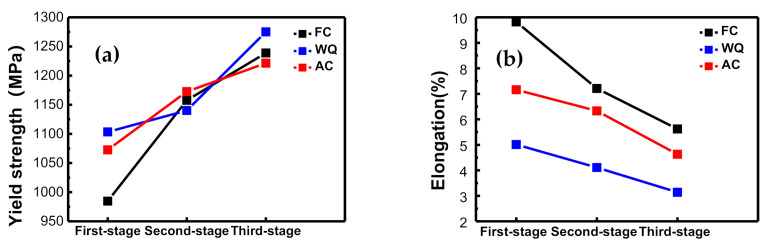
Yield strength (**a**) and elongation (**b**) of samples after aging treatment.

Figure 7 and Table 2 show the mechanical performance the samples after third-stage aging treatments. After three heat treatments, the tensile strength and yield strength of the samples increased gradually, while the plasticity and impact toughness decreased gradually no matter what kind of cooling.

In the first solution stage treatment, the yield strength of the sample by WQ was the highest due to the minimum thickness of martensite α (7.6 μm) of WQ samples, which was 1/2 and 1/6 of the thickness of primary lamellar α of AC (24.9 μm) and FC (60.1 μm) samples, respectively. There were more α/β phase interfaces after the second solution stage treatment, due to the precipitation of a large number of fine secondary lamellar α phase, and the α/β interfaces more effectively prevented the dislocation movement [22,23]. Therefore, the strength of the samples improved after the second-stage solution processes [24,25]. The WQ samples Q after aging treatment had a higher yield strength (1236.35 MPa) compared to the FC and AC samples, which was mainly attributed to the fact that the WQ samples had a faster cooling rate and retained more metastable β phases, resulting in the generation of a large number of dispersed α phase during aging treatment. The dispersed α phase can hinder the dislocation slip and delay the crack nucleation and reduce the crack growth rate [26,27].

In the first solution stage treatment, the wide α clusters (31 μm) led to the good elongation (9.82%) of the FC samples because the α clusters provided adequate space for the slip of dislocations [28]. In the second solution stage and third solution stage treatment, the content of primary lamellar α (second-stage: 19.8 vol.%, third-stage: 11.0 vol.%) of the FC samples was lower than that of the AC and WQ samples. The elongation of the FC samples (second-stage: 7.21%, third-stage: 5.62%) was better than that of the AC and WQ samples. The β phase effectively reduced the effective distance of the dislocation motion [29]. The higher relative content of the β phase as conducive to improving plasticity [29,30,31]. Therefore, after the aging treatment, the FC samples had better comprehensive mechanical properties than the AC and WQ samples.

**Figure 8:**
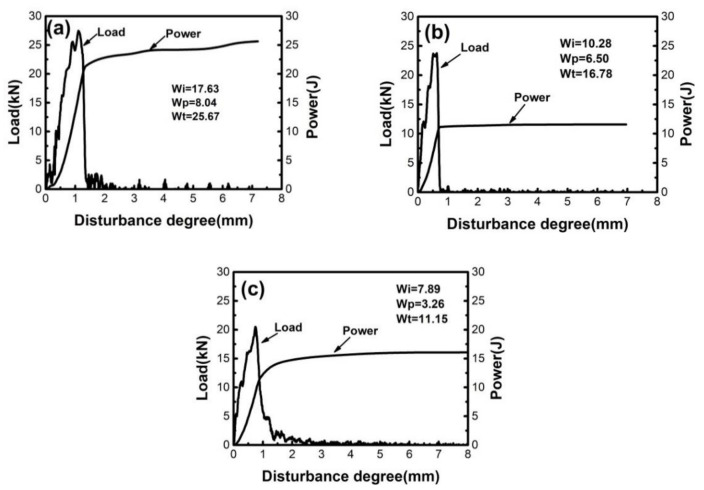
Load-disturbance curve of impact samples after the first solution stage treatment (**a**) FC; (**b**) AC; (**c**) WQ.

In order to study the toughness of the samples cooled at different rates after the first and third solution stage treatment, the impact tests were carried out. Figure 8 shows the load-disturbance curves of impact samples with different cooling modes after the first-stage solution treatment. Wi, Wp, and Wt represent the energy required for crack initiation, crack propagation, and total energy, respectively. The total fracture energy of the material was composed of the energy consumed in the crack initiation and crack propagation stages, so the shape of the oscillographic impact curve was determined by the amount of energy absorbed in each stage. It can be found in Table 1 and Figure 8 that the total fracture energy of FC samples (25.67 J) during the first solution-stage treatment was greater than the AC samples (16.78 J) and WQ samples (11.15 J). In the first solution stage treatment, the impact toughness decreased with the increase of the cooling rate, which was consistent with the evolution of ductility. After the aging treatment, the impact toughness of the samples decreased compared with the first solution stage treatment.

**Figure 9:**
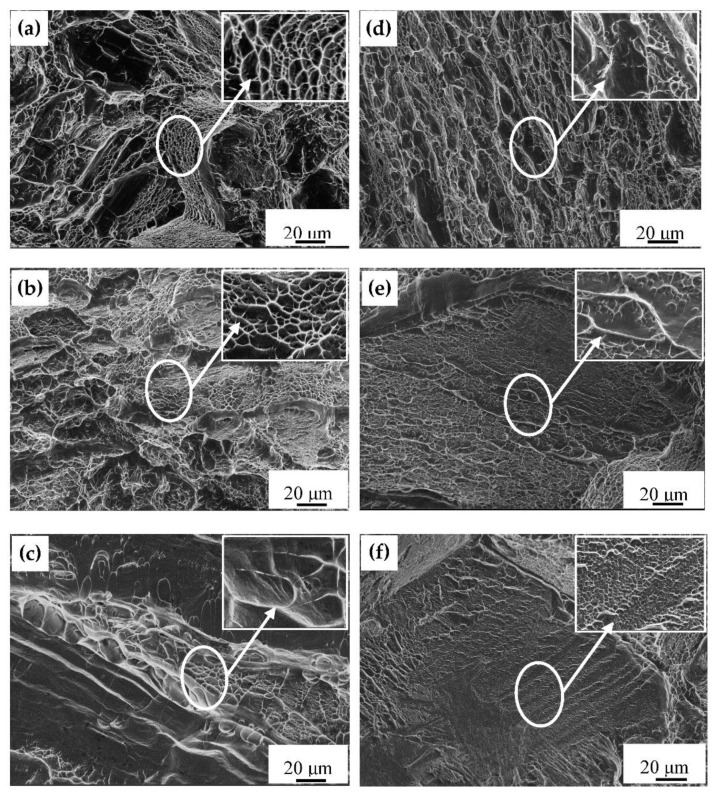
Fracture morphology of samples after the first solution stage treatment (**a**–**c**) and aging treatment (**d**–**f**): (**a**,**b**) FC; (**c**,**d**) AC; (**e**,**f**) WQ.

The fracture morphology of the samples after the first and the third treatment are shown in Figure 9. There were more dimples and a small amount of tearing edges in the sample fracture after the first solution stage treatment (Figure 9a–c), indicating a mainly ductile fracture. As the cooling rate increased, the number of dimples gradually decreased, and more tearing edges appeared. There were a few shallow and elongated dimples on the tearing edge in the fracture of the WQ samples (Figure 9c). Compared to the first solution stage treatment, the number of dimples of the FC samples after aging treatment (Figure 9d) significantly decreased, along with the increase in the number of the tearing edges; the fractures of AC (Figure 9e) and WQ samples (Figure 9f) after aging treatment present a large number of torn edges and cleavage planes. The ductility decreases compared with that of the FC samples.

## 4. Conclusions

The cooling rate of the first solution stage was found to significantly impact the thickness and quantity of primary lamellar α or martensite α’. There were apparent clusters in FC and AC samples. The size and amount of the secondary lamellar α increased after the second solution stage treatment compared with the first solution stage treatment. After aging treatment, a large amount of dispersed α phase precipitated on the β matrix, especially for the WQ samples.

The sample was cooled by FC, AC, and WQ in the first solution stage. The tensile strength and yield strength of the samples gradually increased, while the ductility and impact toughness of the samples decreased. The FC samples had the best ductility and impact toughness.

## Figures and Tables

**Table 1:** The thicknesses and volume percentages of primaryα phase after different stage heat treatments.

Heat Treatments	First Solution Stage		Second Solution Stage		Third Solution Stage	
Cooling Way	Thickness/μm	Volume Percentage/%	Thickness/μm	Volume Percentage/%	Thickness/μm	Volume Percentage/%
FC	0.64	60.1	0.92	19.8	1.01	11.0
AC	0.26	24.9	0.78	29.4	0.89	25.7
WQ	0.11	7.6	0.73	23.1	1.19	12.4

**Table 2:** Mechanical properties of samples after different stage heat treatments.

Heat Treatment		Tensile Strength σ_b_/MPa	Yield Strength σ_s_/MPa	Reduction of Area Ψ/%	Elongation δ/%	Impact Energy A/J
First solution stage (990 °C/30 min)	FC	984.65	960.13	13.52	9.82	25.67
	AC	1072.58	1059.45	8.63	7.66	16.78
	WQ	1103.37	1083.23	8.21	5.01	11.15
Second solution stage (900 °C/45 min/AC)	FC	1157.66	1145.11	8.42	7.21	-
	AC	1172.62	1158.45	7.65	6.83	-
	WQ	1140.19	1121.9	8.30	4.11	-
Third aging stage (590 °C/4 h/AC)	FC	1238.80	1236.35	6.13	5.62	14.50
	AC	1221.34	1211.34	6.33	5.13	13.98
	WQ	1275.02	1214.87	6.72	3.14	11.52

## Data Availability

The data presented in this study are available on request from the corresponding author.
